# Oral–Gut Microbiome Axis in Gastrointestinal Disease and Cancer

**DOI:** 10.3390/cancers13092124

**Published:** 2021-04-28

**Authors:** Se-Young Park, Byeong-Oh Hwang, Mihwa Lim, Seung-Ho Ok, Sun-Kyoung Lee, Kyung-Soo Chun, Kwang-Kyun Park, Yinling Hu, Won-Yoon Chung, Na-Young Song

**Affiliations:** 1 Department of Applied Life Science, The Graduate School, Yonsei University, and BK21 FOUR Project, Yonsei University College of Dentistry, Seoul 03722, Korea; SYPARK87@yuhs.ac (S.-Y.P.); SC4610JT@yuhs.ac (B.-O.H.); OSHO94@yuhs.ac (S.-H.O.); 2 Department of Oral Biology, Yonsei University College of Dentistry, Seoul 03722, Korea; MHLIM2020@yuhs.ac (M.L.); LPLUTO@yuhs.ac (S.-K.L.); BIOCHELAB@yuhs.ac (K.-K.P.); 3 College of Pharmacy, Keimyung University, Daegu 42601, Korea; chunks@kmu.ac.kr; 4 Cancer and Inflammation Program, Center for Cancer Research, National Cancer Institute, National Institutes of Health, Frederick, MD 21702, USA; huy2@mail.nih.gov; 5 Department of Oral Biology, Oral Cancer Research Institute, and BK21 FOUR Project, Yonsei University College of Dentistry, Seoul 03722, Korea; WYCHUNG@yuhs.ac

**Keywords:** oral microbiome, gut microbiome, oral–gut microbiome axis, GI disease, GI cancer

## Abstract

**Simple Summary:**

Microbiota dysbiosis is correlated with numerous diseases in the human body. To date, the research on the microbiome-associated diseases been focused on an organ-specific microbiome. However, the interorgan microbial network is emerging as an important regulator in physiological functions and pathological processes. The oral cavity and gut are the two largest microbial ecosystems. Recent studies have demonstrated that the oral-to-gut and gut-to-oral microbial transmission can regulate pathogenesis, indicating the presence of the oral–gut microbiome axis. Here, we will review the role of the oral–gut microbiome axis in gastrointestinal disease and cancer, which may provide insight for precise diagnosis/prognosis and effective treatment.

**Abstract:**

It is well-known that microbiota dysbiosis is closely associated with numerous diseases in the human body. The oral cavity and gut are the two largest microbial habitats, playing a major role in microbiome-associated diseases. Even though the oral cavity and gut are continuous regions connected through the gastrointestinal tract, the oral and gut microbiome profiles are well-segregated due to the oral–gut barrier. However, the oral microbiota can translocate to the intestinal mucosa in conditions of the oral–gut barrier dysfunction. Inversely, the gut-to-oral microbial transmission occurs as well in inter- and intrapersonal manners. Recently, it has been reported that oral and gut microbiomes interdependently regulate physiological functions and pathological processes. Oral-to-gut and gut-to-oral microbial transmissions can shape and/or reshape the microbial ecosystem in both habitats, eventually modulating pathogenesis of disease. However, the oral–gut microbial interaction in pathogenesis has been underappreciated to date. Here, we will highlight the oral–gut microbiome crosstalk and its implications in the pathogenesis of the gastrointestinal disease and cancer. Better understanding the role of the oral–gut microbiome axis in pathogenesis will be advantageous for precise diagnosis/prognosis and effective treatment.

## 1. Introduction

The microorganisms in and on the human body outnumber the human cells by at least 10-fold [1]. The human microbiome is remarkably diverse even between healthy individuals [2]. In an individual, each microbial habitat shows a distinct pattern of microbial populations [3]. In an effort to characterize human microbial communities, the first phase human microbiome project (HMP) launched in 2007 and has analyzed microbial communities of 300 healthy adults, including 15 body sites in men and additional three vaginal sites in women [3,4]. Since the HMP has completed, a new era of “microbiome” has begun with breakthrough discoveries on the relationship between microbiomes and human health [5,6,7,8]. Currently, the human microbiome is emerging as an important regulator in the human physiology.

Gut and oral microbiomes are the two largest microbial ecosystems in the human body [9]. Based on the HMP, among the 15 different body habitats, oral and fecal microbiomes are ecologically rich and taxonomically diverse [3]. It is noteworthy that the oral cavity and gut are linked physically as well as chemically. However, most of the research on the oral and gut microbiomes has been conducted separately in an organ-specific manner, rather than in an integrative context. The latest studies have proven the involvement of microbiome in the interorgan networks, such as the gut–brain and gut–lung axes [10,11]. In this regard, the intestinal colonization of oral microbiota and fecal–oral transmission have been reported to frequently occur and modulate pathophysiological processes in the human body [12,13,14]. Here, we will highlight the oral–gut microbiome axis and its implications in the health and disease of the gastrointestinal (GI) system.

## 2. Oral and Gut Microbiomes: Connection and Segregation

### 2.1. Oral Cavity and Gut: Connected through GI Tract

The human digestive system consists of the GI tract and the accessory digestive organs, including liver and pancreas. The GI tract is well-lined by the mucous membrane, beginning at the mouth and ending at the gut—more precisely, the anus. Thus, the oral cavity and gut are anatomically continuous regions connected through the GI tract. Moreover, both sites are also chemically connected, since saliva and digested food pass through the GI tract [13]. Generally, the GI tract is thought to be external to the body due to the hollow canal structure. The oral cavity, a gateway of the digestive tract, is directly exposed to the outside environment, such as microorganisms, nutrients and other xenobiotics. In this regard, both oral cavity and gut provide a proper environment for divergent microbes to thrive. The HMP has revealed that more than half of bacteria in the human body reside in the GI tract (29%) and the oral cavity (26%) [9]. In addition to this abundance, the oral and gut microbiomes are highly diverse and concomitantly show unique signatures distinguished from each habitat [3].

### 2.2. Oral Microbiome Composition

According to the human oral microbiome database (HOMD), the oral cavity presents approximately 700 species of microorganisms (from the HOMD website; www.homd.org; accessed on 20 January 2021). Commensals in the oral cavity contain *Firmicutes*, *Proteobacteria*, *Bacteroidetes*, *Actinobacteria*, *Fusobacteria*, *Neisseria*, and TM7 at the phylum level [15,16,17]. The oral cavity has several distinct microbial habitats, including buccal mucosa, subgingival plaque, supragingival plaque, keratinized gingiva, hard palate, saliva, tonsil, tongue, and throat. The buccal and palatal mucosae showed lower diversity than other oral habitats [18]. Regardless of the niche location, all the oral sites of the healthy subject carry *Streptococcus*, *Gemella*, *Veillonella*, *Haemophilus*, *Neisseria*, *Porphyromonas*, *Fusobacterium*, *Actinomyces*, and *Prevotella* at the genus level [19,20]. In addition to these common bacterial clades, each niche has a well-differentiated bacterial composition. Based on the microbial community structure, the oral niches can be divided into three distinct groups as follows: Group 1—buccal mucosa, keratinized gingiva, and hard palate; Group 2—saliva, tongue, tonsils, and throat; Group 3—sub- and supragingival plaque [20]. This segregation of the oral microbiome by niches is plausibly attributed to several factors, such as pH, salinity, redox potential, oxygen, and nutrition [15,21]. Moreover, dental hygiene is another important factor that shapes the oral microbiome, since the oral cavity is directly open to the outside environment [22,23].

### 2.3. Gut Microbiome Composition

The gut is the largest and the most well-characterized microbial ecosystem in the human body, which harbors about 500 to 1000 species in more than 50 different phyla [24]. The gut microbiota, mostly anaerobes, is composed of five major phyla—*Bacteroidetes*, *Firmicutes*, *Actinobacteria*, *Proteobacteria*, and *Verrucomicrobia*—but dominated by two phyla—*Bacteroidetes* and *Firmicutes*, which account for more than 90% [25]. At the genus level, *Bacteroides* is the most abundant [26]. The human gut microbiota is known to be established early in life and can then be changed by age and environments, such as diet and nutrition, similar to the human oral microbiome [27,28]. Thus, both oral and gut microbiomes directly reflect the health status of the host.

Although the gut is continuously linked to the oral cavity, the gut microbiota composition can be distinguished from the one of oral cavity. At the phylum level, the oral cavity is dominated by *Firmicutes*, while the stool microbiota is mostly abundant with *Bacteroidetes* [20]. This segregation could be attributed to gastric acid in the stomach and bile acids in the duodenum [29,30]. It has been reported that long-term usage of proton pump inhibitors (PPIs) increases risk of enteric infection [31,32]. Of note, low gastric acidity by PPIs can reduce the diversity of gut microbial ecosystem and change the gut microbiome composition [33]. Moreover, bile acids can induce damage on the membrane and/or DNA integrity of enteric bacteria, acting as a potent antimicrobial barrier between oral cavity and gut [34,35]. Thus, gastric acidity and bile acid pool are responsible for distinctive patterns of the gut and oral microbiomes.

### 2.4. Physiological Functions of Gut Microbiome: Lessons from Germ-Free Mice

The human gut microbiome profiles can be shifted depending on health status, environmental factors, genetics, and even life styles [2]. The metagenomic analysis has revealed that the human gut-resident bacterial community regulates metabolic pathways, such as carbon metabolism and amino acid synthesis [26]. Microorganisms display conserved molecular motifs known as microbial-associated molecular patterns and pathogen-associated molecular patterns (PAMPs), which can be recognized by the host through pattern recognition receptors (PRRs), such as toll-like receptors [6,36]. This microbial–host interaction can stimulate the immune system and inflammatory responses in the human [36]. This means that the gut microbiota can modulate central biological functions, metabolism and immunity in the human body, and thus gut dysbiosis is associated with numerous human diseases, from infectious disease to Alzheimer’s disease [5,10,37]. However, it is challenging to prove whether the gut microbiota is the cause or consequence of the human health status.

Alternatively, germ-free (GF) animals have provided insightful clues for the physiological functions of the gut microbiome [38,39]. Compared to specific pathogen-free (SPF) mice, GF mice have reduced intestinal mass, shorter villi, and decreased total surface area of the small intestine, indicating defects in GI tract development [38]. In line with this, GF mice show metabolic abnormalities, such as altered cholesterol metabolisms and reduced intestinal amounts of short-chain fatty acids, one of the important energy sources [40,41]. GF mice thus display lower body fat contents and resistance to high fat diet-induced body weight gain, compared to SPF mice [42,43]. However, the body fat contents were restored by conventionalization of GF mice via applying cecal contents of SPF mice [43]. In aspects of the immunity, GF mice have defects in the development of Peyer’s patches and mesenteric lymph nodes, reduced numbers of CD4, CD8, and Foxp3 T cells, and decreased production of secretory immunoglobulin A in B cells [6]. These disorders can be recovered by microbiota reconstitution through either cohousing with SPF mice or oral inoculation of fecal contents from SPF mice [44,45,46]. Taken together, it is evident that the gut microbiota plays a crucial role in maintaining physiological homeostasis, primarily metabolism and immunity.

### 2.5. Physiological Functions of Oral Microbiome: Local and Systemic Effects

Although the oral cavity is the second largest microbial habitat in the human body, the cumulative knowledge is not sufficient to fully understand the implications of oral microbiome in the human health. It is unquestionable that the oral microbiome is directly associated with dental health [23,47]. There are well-identified keystone pathogens in oral diseases, such as *Streptococcus mutans* for dental caries and *Porphyromonas gingivalis* for periodontitis [48,49]. Moreover, patients with oral squamous cell carcinoma showed alteration in oral microbiome compared to the healthy subjects [50,51]. Based on the oral microbiota analysis, *Fusobacterium* was enriched in oral squamous cell carcinoma (OSCC) patients at the genus level [52,53]. In GF mice, inoculation with oral microbiome promoted chemical-induced oral carcinogenesis, further supporting the direct involvement of oral microbiome in oral disease [54]. Thus, oral microbes modulate dental pathophysiology in a single key pathogen-dependent manner as well as a collective manner.

**Figure 1:**
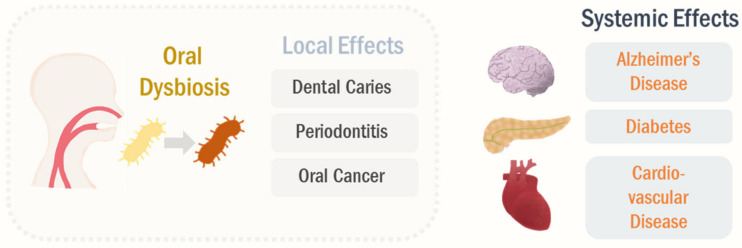
Local and systemic effects of oral microbiome. The oral dysbiosis can regulate the pathological processes in the oral cavity, such as dental caries, periodontitis, and OSCC. The altered oral microbiota profiles can further modulate systemic diseases, including Alzheimer’s disease, diabetes, and cardiovascular disease, beyond the local impacts.

The oral microbiome can affect systemic health conditions, not limited to the dental health (see Figure 1) [55,56]. Epidemiological and experimental evidence supports that oral dysbiosis is closely associated with systemic diseases, including Alzheimer’s disease, diabetes, and cardiovascular disease [57,58,59]. In line with this, the oral microbiota profile was significantly altered in Alzheimer’s disease, such as prevalence of the genera *Moraxella*, *Leptotrichia*, and *Sphaerochaeta* [60]. The oral dysbiotic shifts were associated with the progression of Alzheimer’s disease [61]. Patients with type I diabetes showed higher abundance of the phyla *Actinobacteria* and *Firmicutes* compared to healthy controls [62]. Moreover, the enrichment of the genus *Anaeroglobus* has been reported in the oral micro-biome of patients with symptomatic atherosclerosis [63]. In case of periodontitis, an oral dysbiotic disease, its signature pathogen *P. gingivalis* infection can induce chronic inflammation locally as well as systemically [64,65]. Moreover, oral dysbiosis can induce production of PAMP signals, such as lipopolysaccharide (LPS), resulting in systemic stimulation of innate immune responses and inflammatory transcription factors, including nuclear factor κB [66,67]. These systemic inflammation and immune responses are thought to be one of the primary mechanisms, underlining that the oral microbiome regulates pathogenesis in distal organs.

Notably, oral microbiota can translocate to the other organs, which is considered as another mechanism of oral dysbiosis-induced systemic disease [68,69]. The oral pathogen *P. gingivalis* has been detected in the brain tissues of short-term postmortem Alzheimer’s disease patients [70]. The direct translocation of oral pathogen to brain can aggravate Alzheimer’s disease through inducing neuroinflammation and neurodegeneration [71,72]. Moreover, a number of oral commensal bacteria was detected in atherosclerotic plaques of coronary artery disease patients, further indicating the possible translocation of oral bacteria to the distal organs [73]. The migration of oral microbes can occur more frequently towards the GI system, due to the physical and chemical connections. In certain pathogenic conditions, some of the oral bacterial taxa are colonized and enriched in pancreas and gut, indicating the direct crosstalk between oral and gut microbiotas [74,75,76]. Thus, we will discuss the interaction between oral and gut microbiomes and its pathophysiological functions in the following sections.

## 3. Interconnection between Oral and Gut Microbiomes: Oral–Gut Microbiome Axis

### 3.1. Oral-to-Gut Microbial Translocation

The oral and gut microbiomes are well-segregated due to the presence of the oral–gut barrier, physical distance as well as chemical hurdles, such as gastric acid and bile [20,30,77]. However, the impairment of the oral–gut barrier can allow interorgan translocation and communication. In general, neonates and elderly people have immature or less functional barriers in the body [78,79]. *Bifidobacterium* is the most abundant bacterial genus in the neonatal gut [80]. Interestingly, the gut-resident *Bifidobacterium* has been detected in the oral fluid of neonates [81]. Likewise, elderly people showed prevalence of the oral bacteria in the gut compared to healthy adults, such as *Porphyromonas*, *Fusobacterium*, and *Pseudoramibacter* [82,83]. Moreover, low gastric acidity shifted the gut microbiota composition towards the oral microbiome, further indicating translocation of oral microbiota to gut under the oral–gut barrier dysfunction [33]. Li et al. have demonstrated in vitro that the oral microbiota can invade into the gut and reshape the gut microbial community by cohousing GF mouse groups introduced with human fecal and salivary microbiotas, respectively [84]. Taken together, these data suggest that the oral microbes can overcome the physical and/or chemical barriers between the oral cavity and gut under certain circumstances and potentially translocate into the gut.

Notably, typical oral-resident species have been detected under pathological conditions in the GI tract [74,75,85]. For instance, patients with inflammatory bowel disease (IBD) had significant enrichment of *Haemophilus* and *Veillonella* in the gut mucosa, which are known to be oral commensal microbes [86]. In patients with colon cancer, their gut microbiomes contained several oral taxa, including *Fusobacterium* [87]. This implies that the normal human oral microbiota can invade and colonize in the gut mucosa and become an opportunistic pathogen in conditions of disrupted mucosal homeostasis.

However, this oral–fecal transmission can occur under the physiological conditions as well, not only in pathological contexts or barrier disruption. When HMP consortium data were partitioned into community types for each body site, the oral and gut microbiome types show strong association, even though they were taxonomically different [3,88]. Among the salivary bacteria, *Prevotella* was abundantly found in the stool samples [88]. In line with this, several genera were concomitantly detected from both oral and stool samples of the same healthy subject [20]. By analyzing 310 species from oral and fecal microbiomes in 470 individuals, 125 species were prevalent in both saliva and stool samples, including strains of *Streptococcus*, *Veillonella*, *Actinomyces*, and *Haemophilus* [13]. Taken together, it is obvious that the oral microbiota can translocate into the gut more extensively than expected even in the healthy states, not only in the pathological circumstances.

### 3.2. Fecal-to-Oral Microbial Translocation

Enteric microorganisms can be transmitted by fecal–oral routes through direct contact or indirect exposure via contaminated fluids and foods [89]. The human hand microbiota profile was highly overlapped with oral and gut microbiome patterns, suggesting that the human hand is a carrier for fecal-to-oral microbial transmission [14]. Thereby, the fecal–oral route of microorganisms has been frequently reported in developing countries, due to poor hygienic status, such as lack of clean water supply and public health system [90,91]. Furthermore, immunocompromised individuals are susceptible to fecal–oral transmission as well. In case of head and neck cancer patients, radiation therapy was highly associated with oral colonization of gram-negative enteric rods, which can be further exacerbated by poor oral hygiene conditions [92,93]. Thus, poor hygienic and/or immunocompromised conditions can facilitate the fecal–oral route in the same individuals.

**Figure 2:**
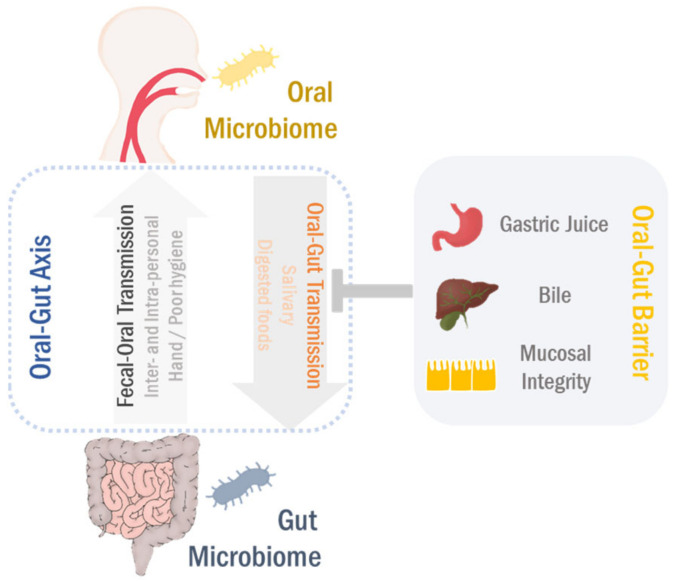
Oral–gut microbiome axis. The oral microbiota can translocate to the gut in conditions of the oral–gut barrier disruption. Likewise, the gut microbes transmit to the oral cavity in both intra- and interpersonal manners, particularly related to poor hygienic conditions. This bidirectional interaction between oral and gut microbiomes develops the microbial ecosystems in both habitats through either competition or cooperation, eventually regulating the pathophysiological processes in the gastrointestinal (GI) tract.

In addition to intrapersonal transmission, the fecal–oral route is considered as an important mechanism for human-to-human transmission of pathogens as well. Enteric viruses, such as hepatitis A virus (HAV) and hepatitis E virus (HEV), are well-known to transmit through the fecal–oral route and thus easily spread by person-to-person contact, particularly in insanitary conditions [94,95,96]. The enteric viruses can interact with the gut microbiota in both direct and indirect manners, resulting in devastating effects on the gut microbial ecosystem [97,98]. HEV infection has been reported to increase the abundance of *Lactobacillaceae* and *Gammaproteobacteria* in the fecal samples of patients with acute liver failure [99]. In contrast, supplementation with the probiotic bacterium *Enterococcus faecium* NCIMB 10415 can effectively promote HEV removal in infected pigs [100]. In addition to enteric viruses, *Helicobacter pylori*, major causal bacteria of severe gastroduodenal diseases, can transmit via the fecal–oral route as well, showing a correlation with HAV infection [101,102]. Although further investigation is required to understand the role of the fecal–oral transmission in oral and gut microbiomes, it is convincing that the oral and gut microbiomes are closely connected through both oral-to-gut and fecal-to-oral routes (see Figure 2). This bidirectional interaction can mutually shape and/or reshape the microbial ecosystem of both habitats, finally modulating physiological and pathological processes in the GI system. Thus, both oral–gut and fecal–oral directions will collectively be referred to as the “oral-gut microbiome axis” in the following sections.

## 4. Oral–Gut Microbiome Axis in Human GI Diseases and Cancers

### 4.1. Inflammatory Bowel Disease

IBD represents chronic inflammatory disorders of the colon and small intestine, including Crohn’s disease (CD) and ulcerative colitis (UC). IBD is thus strongly associated with the gut microbiome dysbiosis. The gut microbiomes of IBD patients show reduced diversity and shifts in bacterial composition, including loss of the bacterial phylum *Firmicutes* and increased abundance of the phyla *Proteobacteria* and *Bacteroidetes* [103,104,105,106]. These dysbiotic events have been observed more profoundly in intestinal mucosal tissue biopsies, rather than in the stool [86]. On the intestinal mucosal surface, bacterial invasion and biofilm formation were frequently detected in IBD patients compared to the healthy subjects, indicating that gut barrier dysfunction is involved in IBD pathogenesis [13,107,108].

In healthy states, the gut microbiome is barely invaded and colonized by microbes derived from other habitats, due to the intact mucosal barrier [109]. However, IBD patients manifest increased gut epithelial permeability due to impaired mucosal barrier [110,111]. It is noteworthy that the oral-resident bacterial strains are isolated from the gut microbiome of IBD patients, possibly due to the gut leakiness. *Fusobacterium nucleatum* resides commonly in the oral cavity but rarely in the guts of healthy individuals [112]. Interestingly, IBD patients showed colonization of *F. nucleatum* in the gut, which was more invasive than other *F. nucleatum* strains, indicating the presence of the oral–gut microbiome axis in IBD patients [74,75]. This has been confirmed in vitro by transplantation of the oral microbiota into animal models. In rats, *F. nucleatum*-gavage led to shifts in the gut microbiome and aggravated visceral hypersensitivity [113]. Moreover, salivary microbiota of CD patients successfully colonized in the gut of GF mice [114]. *Klebsiella* was the most prevalent colonizer, which can promote Th1 cell induction and inflammation in the intestine, the key events in IBD pathogenesis [114]. These results further support that oral microbiota, either commensal or pathobiont, can be transmitted into the intestine, promoting IBD pathogenesis via gut dysbiosis.

Hence, oral dysbiosis can directly modulate pathogenesis of IBD by recruiting the oral–gut axis. Periodontitis, a chronic inflammatory oral disease, is strongly associated with alteration of oral microbiota—in particular, with the overgrowth of its keystone pathogen *P. gingivalis* [48,115]. In C56BL/6 mice, oral administration of *P. gingivalis* attenuated the intestinal barrier function via downregulating tight junction proteins, leading to significant alteration of gut microbiome, including enrichment of the family *Clostridiaceae* [116,117,118,119]. Moreover, *P. gingivalis*-inoculated mice showed intestinal as well as systemic inflammation, which can be mediated by *P. gingivalis*-derived endotoxins, such as LPS [117,118,120,121]. Consistent with animal experiments, the meta-analyses have demonstrated that periodontitis is strongly associated with two major forms of IBD, CD, and UC, respectively [122,123]. Taken together, the oral pathogen(s) can interfere with intestinal barrier function and invade the gut mucosa, which induces the intestinal dysbiosis and chronic inflammation, consequently leading to IBD pathogenesis. Notably, IBD patients as well as colitis-induced mice displayed alterations in their salivary microbiota compositions, which were associated with inflammatory responses, indicating that the oral–gut microbial interactions could be bidirectional [124,125].

### 4.2. Colorectal Cancer

Colorectal cancer (CRC) is one of the most common cancer types and the second leading cause of cancer mortality worldwide [126]. IBD is the most well-established risk factor for development and progression of CRC [127]. Thus, IBD and CRC share etiological factors in pathogenesis, including distinct changes in the gut microbiome [128,129]. Similar to IBD, CRC is strongly associated with gut dysbiosis. CRC patients show the distinct patterns of microbial compositions in both fecal and intestinal mucosal samples compared to healthy individuals [87,130,131,132]. Consistently, profound alterations of gut microbiota have been found in both colitis-associated and chemical-induced CRC mouse models, supporting the relationship between gut dysbiosis and CRC [133,134]. Research using GF mice has further demonstrated that alteration of gut microbiota can directly facilitate inflammation-associated CRC development [135,136].

Interestingly, several oral taxa have been detected in the gut of CRC patients, which include *Parvimonas*, *Peptostreptococcus*, and *Fusobacterium*, indicating the presence of the oral–gut microbiome axis in CRC [87]. Among these oral resident bacteria, *F. nucleatum* was prevalent in tumor tissues and feces of CRC patients compared to the healthy subjects, which is consistent with IBD [87,137,138,139]. In mouse colitis models, oral administration of *F. nucleatum* induced inflammation as well as tumorigenesis in the small and large intestines [139]. *F. nucleatum* seems to readily attach to the host CRC cells that express endothelial cadherin, and then stimulate proinflammatory responses and cell proliferation [138,139]. Similar to IBD, colorectal tumors showed impaired intestinal barrier functions, which might explain the intestinal colonization of oral microbiota [140,141].

Moreover, it has been reported that *F. nucleatum* can coaggregate and coinfect with the oral pathogen *P. gingivalis* [142,143]. Despite the limitations of in vitro approaches, *P. gingivalis* invaded CRC cells and promoted cancer cell proliferation, indicating the involvement of periodontal pathogen in colorectal tumorigenesis [144]. In line with this, *P. gingivalis* serum antibody levels were positively correlated with mortality in CRC patients [145]. Furthermore, a meta-analysis demonstrated that periodontitis is associated with increased CRC risk [146,147]. Taken together, these investigations serve as evidence of the association between oral dysbiosis, oral–gut microbiome axis, and CRC pathogenesis.

### 4.3. Chronic Liver Disease

Cirrhosis is a late-stage liver disease caused by chronic liver disorders, such as nonalcoholic fatty liver disease (NAFLD) and nonalcoholic steatohepatitis (NASH) [148]. Interestingly, a broad spectrum of chronic liver diseases has been related to intestinal dysbiosis [149,150,151,152,153]. Patients with NAFLD, NASH, or cirrhosis showed significant increase in the phylum *Proteobacteria* in their stool samples compared to the healthy controls, indicating the association of the gut microbiome in hepatopathogenesis [150,151,152]. In this regard, GF mice were protected from high fat diet-induced lipid accumulation in the liver, compared to SPF mice [41]. Moreover, GF mice colonized with NAFLD-prone gut microbes developed severe hepatic steatosis, further supporting that the gut dysbiosis can be a direct causal of chronic liver diseases [154].

Since the gut and liver are physically connected by the biliary tract and portal vein, gut microbes can translocate to the liver if the mucosal barrier is impaired [155]. Bile acids possess antimicrobial activities and circulate between gut and liver for recycling, dual-functioning as a barrier as well as a bridge [34]. Chronic liver diseases are often related to defective formation and/or secretion of bile acids, which can increase the intestinal permeability [156,157]. Thereby, biliary obstruction facilitates bacterial translocation from gut to liver [158,159]. In gallstone patients, the microbial composition was shifted in both biliary tract and gut compared to the normal controls, such as enrichment of *Proteobacteria*, supporting the presence of the gut–liver microbiome axis in chronic liver diseases [160].

By converging with the gut–liver microbial crosstalk, the oral–gut microbiome axis is emerging as an important modulator in chronic liver diseases. It is noteworthy that the metagenomic analysis has proven invasion and colonization of oral commensals in the gut of patients with cirrhosis [152]. Another study also showed the enrichment of oral microbes in the gut of cirrhotic patients with alcohol dependence [161]. These data support that intestinal transition of oral microbes is associated with cirrhosis; however, the underlying mechanism is not clear yet. As mentioned earlier, PPI promoted oral-to-gut microbial transition due to low gastric acidity [33]. Likewise, PPI treatment altered the gut microbiota composition in cirrhotic patients, particularly displaying overgrowth of oral-resident bacteria in the gut [162]. The same research group has demonstrated the concomitant changes of salivary and stool microbiomes in cirrhosis, further suggesting that the oral–gut microbiome axis regulates hepatic pathogenesis [163].

Oral dysbiosis thus potentially aggravates chronic liver diseases via shifts in the gut microbiome. Indeed, periodontitis is significantly associated with NASH, NAFLD, and cirrhosis [164,165,166,167]. *P. gingivalis*, a periodontal keystone pathogen, has been detected in oral samples from NAFLD and viral infection-related cirrhosis patients [168,169]. In high fat diet-fed mice, odontogenic infection by *P. gingivalis* facilitated progression of NAFLD and NASH through lipid accumulation, fibrosis, and inflammation in the liver [170,171]. Overall, oral dysbiosis can exacerbate chronic liver diseases, possibly through modulation of the gut ecosystem. Concomitantly, oral dysbiosis might reflect the intestinal dysbiotic ecosystem driven by hepatic diseases.

### 4.4. Hepatocellular Carcinoma

Hepatocellular carcinoma (HCC) is developed through a stepwise progression, from NAFLD/NASH to cirrhosis and finally to HCC [172]. In a mouse hepatocarcinogenesis model, SPF mice were more susceptible for HCC development than GF mice, similar to the chronic liver diseases [173]. In a gnotobiotic mouse model, certain types of intestinal bacteria, such as *Escherichia coli* and *Streptococcus faecalis*, can significantly increase liver tumorigenesis, indicating the direct involvement of the gut microbiota in HCC pathogenesis [174]. In line with this notion, HCC patients showed decrease in butyrate-producing genera, such as *Ruminococcus*, *Oscillibacter*, *Faecalibacterium*, *Clostridium* IV, and *Coprococcus*, while an increase in LPS-producing genera, including *Klebsiella* and *Haemophilus* in the stool samples, compared to the healthy controls [175]. Moreover, the level of gut dysbiosis tends to increase with the progression of HCC [176]. In HCC patients with cirrhosis, the fecal microbiota composition was distinguished from that of cirrhosis patient without HCC, such as significant enrichment of *E. coli* and *Fusobacteriia* [177,178]. In a chemically induced HCC mouse model, an enteric bacteria *Helicobacter hepaticus* has been found within tumors, which directly caused HCC development and progression, further supporting that gut dysbiosis can induce HCC pathogenesis [179,180]. However, *H. hepaticus* was not detected in human HCC samples, while the presence of other *Helicobacter* species, such as *H. pylori*, was confirmed [181,182]. Thus, HCC development is strongly associated with gut dysbiosis.

Interestingly, changes of oral microbiota profiles have been reported in HCC patients compared to healthy subjects [183,184,185]. HCC patients showed high abundance of the genera *Haemophilus*, *Porphyromonas*, and *Filifactor* in the salivary microbiota [184]. In HCC patients with cirrhosis, the genera *Oribacterium* and *Fusobacterium* were prevalent based on the microbiome profiles of tongue coat [185]. Moreover, chronic periodontitis was associated with advanced HCC stages, suggesting the correlation between oral dysbiosis and HCC [186]. Of note, *Fusobacterium* has been enriched in both oral and gut microbiomes in HCC patients with cirrhosis, which confers a possibility that the oral microbes can regulate HCC pathogenesis through the oral–gut microbiome axis, but needs further investigation [176,178,185].

### 4.5. Pancreatic Ductal Adenocarcinoma

The pancreas is a part of the digestive system that secretes enzymes to break down lipids, proteins, and carbohydrates. The main pancreatic duct combines with the common bile duct, which both connect to the duodenum. Under normal healthy conditions, the pancreas is thought to be a sterile organ [187]. However, patients with pancreatic ductal adenocarcinoma (PDAC) showed increased abundance of bacteria, such as *Gammaproteobacteria* within tumors and *Enterococcus faecalis* in the pancreatic juice and pancreatic tissues [188,189]. Moreover, intratumoral microbiome diversity was correlated with prognosis of PDAC [190]. With a more integrative view, PDAC patients display the distinct microbiome patterns in the pancreatic tissues, tumors, as well as fecal samples, indicating the involvement of gut–pancreatic microbial crosstalk in PDAC pathogenesis [190,191]. In particular, *Proteobacteria* was concomitantly enriched in both gut and pancreas of PDAC patients [188,191]. In experimental mice, increased intestinal permeability was associated with gut-to-pancreatic microbial translocation, which could accelerate PDAC progression [190,191,192]. In the gut microbiome-ablated mice, repopulation with the fecal microbiome from PDAC-bearing mice can significantly promote pancreatic tumorigenesis, indicating the direct contribution of gut microbiome to PDAC progression [191]. Thus, the intestinal microbiome seems to be closely coordinated with the pancreatic microbial ecosystem, which plays a crucial role in PDAC pathogenesis.

Surprisingly, the oral microbiome is also associated with PDAC pathogenesis. Based on meta-epidemiological studies, periodontitis, a major oral dysbiotic disease, can significantly increase the risk and mortality of PDAC [193,194]. In consistent, carriage of its key pathogen, *P. gingivalis,* positively correlates to higher risk and mortality in PDAC patients [145,195,196]. In a mouse PDAC model, oral administration of *P. gingivalis* accelerated cell proliferation and epithelial–mesenchymal transition, finally promoting PDAC progression [197]. Interestingly, intracellular *P. gingivalis* directly promoted tumor cell growth in human pancreatic cancer cell lines [198]. These suggest that oral dysbiosis can be a direct etiology as well as a useful marker for diagnosis and prognosis in PDAC pathogenesis.

In addition to oral dysbiosis, PDAC patients showed a distinct shift in the oral microbiome compared to the healthy subjects [196,199]. Notably, *Fusobacterium*, a well-known oral bacterial group, has been detected in human PDAC tissues, although its relationship with prognosis of PDAC is controversial [196,200]. Moreover, the pancreatic microbiome was highly overlapped with the intestinal microbiome in PDAC patients [85]. Both pancreatic and intestinal microbiomes exhibited the relative abundance of the oral taxa *Fusobacterium* and *Porphyromonas* [85]. Thus, it is plausible that certain types of oral microbes can migrate to the gut and even further to the pancreas, which can promote PDAC pathogenesis through the coordinated modulation of the intestinal and pancreatic microbiomes. In support of this notion, a correlation has been found between oral, intestinal, and pancreatic microbiomes in PDAC patients, particularly coabundance of oral-originated *F. nucleatum* subsp. *vincentii* [76]. Taken together, these data suggest that the oral–gut microbiome axis can modulate PDAC pathogenesis, even further creating the oral–gut–pancreatic microbial route.

## 5. Perspectives

It has been well-appreciated that the gut and oral dysbioses are associated with numerous diseases [5,8,55,56]. To date, most of the research on microbiome-associated diseases have been conducted with respect to a single organ-specific microbiome, with less concern for an interorgan microbial communication. The oral cavity and gut are the two largest microbial habitats in the human body [9]. Cumulative evidence supports that the oral microbiota can change the overall gut microbial ecosystem through direct translocation and/or rather indirectly, by secretomes of oral bacteria [12,201,202]. Gut-to-oral microbial transmission can occur as well, particularly under certain circumstances, such as poor hygienic and immunocompromised conditions [14,90,92]. Collectively, the bidirectional crosstalk between oral and gut microbiomes can develop the oral–gut microbiome axis, which plays a crucial role in regulating pathogenesis of various human diseases, primarily in the GI system (see Table 1, Table 2 and Table 3).

It is noteworthy that the oral–gut microbiome axis improves prediction of pathogenesis and prognosis in the GI system. The meta-analysis has shown that oral microbiome changes are associated with the risk of GI cancer, including CRC, PDAC, and HCC, which can be a potential index for early detection [203]. Farrell et al. have validated PDAC-specific oral microbial patterns as a PDAC biomarker [199]. The concomitant enrichment of two oral bacterial species, *Neisseria elongata* and *Streptococcus mitis*, can specifically distinguish PDAC patients from healthy subjects [199]. In convergence with the coordinated modulation of oral and gut microbiomes, the oral dysbiotic pattern can provide more consolidated information on the pathogenesis. In CRC patients, combining oral and gut microbiome data can significantly increase the sensitivity to predict and detect polyps and/or tumors [68]. Although it is challenging to uncover the causal relationship between the microbiome and disease, integration of oral and gut microbiomes might be helpful to overcome this hurdle.

Furthermore, the oral cavity is highly accessible than the intestine, since it is open to the outside of body. For gut microbiome analysis, the samples are primarily collected from stool and mucosal biopsy [204]. The fecal samples are noninvasive and cost-effective, but can be contaminated with urine and bring unpleasant feelings to the sample donor [204,205]. However, the biopsy sampling is invasive and not suitable for healthy individuals, while it can generate more accurate data [204]. In case of the oral microbiome analysis, the samples can be obtained from cotton swabbing, saliva, and mouth-rinse [4,206]. Compared to sampling methods for gut microbiota, oral microbiota collection is practically more convenient and available regardless of the health status, without any invasion or hygienic issues. Thus, in conjunction with the gut microbiome, the oral microbiome further provides feasible merits as a diagnostic/prognostic tool as well as a therapeutic target. Moreover, the modification of oral microbiome simply by improvement of dental hygiene and/or supplementation with probiotics can modulate the pathogenesis of disease [47,207].

## 6. Conclusions

Taken together, it is evident that the oral–gut microbiome axis is strongly associated with diseases in the GI system. Understanding the correlation of the oral–gut microbiome axis in pathogenesis confers an advantage for precise diagnosis/prognosis and effective treatment. Thus, integrative research on the interorgan microbial network will shed light on novel strategies to better control microbiome-associated diseases.

## Figures and Tables

**Table 1:** Oral–gut microbiome axis in colon diseases.

Disease	Association with Oral and Gut Microbiomes	References
IBD	*Association with gut microbiome*	
	• Gut dysbiosis in IBD patients	[103,104,105,106]
	*Association with oral microbiome*	
	• Altered salivary microbiome in IBD patients	[124]
	• Altered oral microbiome in colitis-induced mice	[125]
	• Periodontitis was associated with increased IBD risk	[122,123]
	• Oral administration of *P. gingivalis* altered the gut microbiome in mice	[117,119]
	• *F. nucleatum*-gavage altered gut microbiome in rat	[113]
	*Prevalence of oral microbiota*	
	• *F. nucleatum* in the gut microbiome of IBD patients	[74,75]
	• *Klebsiella* in the gut microbiome of GF mice transplanted with salivary microbiota from CD patients	[114]
CRC	*Association with gut microbiome*	
	• Gut dysbiosis in CRC patients	[87,130,131,132]
	• Altered gut microbiome in CRC mouse models	[133,134]
	*Association with oral microbiome*	
	• Periodontitis was associated with increased CRC risk	[146,147]
	• A positive correlation between *P. gingivalis* serum antibody level and mortality in CRC patients	[145]
	• Oral administration of *F. nucleatum* promoted colon carcinogenesis in mice	[139]
	*Prevalence of oral microbiota*	
	• Parvimonas, Peptostreptococcus, and Fusobacterium in the gut of CRC patients	[87]
	• F. nucleatum in tumors and feces of CRC patients	[87,137,138,139]

**Table 2:** Oral–gut microbiome axis in liver diseases.

Disease	Association with Oral and Gut Microbiomes	References
Chronic liver diseases	*Association with gut microbiome*	
	• Gut dysbiosis in NAFLD	[149,150]
	• Gut dysbiosis in NASH	[151,152,153]
	• Gut dysbiosis in cirrhosis	[152]
	*Gut–liver microbiome axis*	
	• Gut-to-liver translocation by biliary obstruction	[158,159]
	• Concomitant shifts in the biliary tract and gut microbiomes in gallstone patients	[160]
	*Association with oral microbiome*	
	• Concomitant shifts in the oral and gut microbiomes in cirrhosis	[163]
	• Periodontitis was associated with NASH, NAFLD, and cirrhosis	[164,165,166,167]
	• Oral administration of *P. gingivalis* accelerated progression of NAFLD and NASH in high fat diet-fed mice	[170,171]
	*Prevalence of oral microbiota*	
	• Colonization of oral bacteria in the gut of cirrhosis patients	[152]
HCC	*Association with gut microbiome*	
	• Gut dysbiosis in HCC patients	[175]
	• Prevalence of *E. coli* and *Fusobacteria* in the gut microbiome of HCC patients with cirrhosis	[177,178]
	*Association with oral microbiome*	
	• Altered oral microbiome in HCC patients	[183,184,185]
	• Prevalence of *Fusobacterium* and *Oribacterium* in the tongue microbiome of HCC patients with cirrhosis	[185]
	• Periodontitis was associate with advanced HCC stages	[186]
	*Prevalence of oral microbiota*	
	• Prevalence of *Fusobacterium* in both oral and gut microbiomes of HCC patients with cirrhosis	[176,178,185]

**Table 3:** Oral–gut microbiome axis in pancreatic disease.

Disease	Association with Oral and Gut Microbiomes	References
PDAC	*Association with gut microbiome*	
	• Gut dysbiosis in PDAC patients	[85,188,190,191]
	*Gut–pancreatic microbiome axis*	
	• Concomitant shifts in the gut, pancreatic, and tumor microbiomes of PDAC patients	[188,190,191]
	• Overlap between gut and pancreatic microbiomes	[85]
	*Association with oral microbiome*	
	• Altered oral microbiome in PDAC patients	[196,199]
	• Concomitant shifts in the oral, gut, and pancreatic microbiomes of PDAC patients	[76]
	• Periodontitis was associated with increased PDAC risk and mortality	[193,194]
	• Carriage of *P. gingivalis* was associated with increased PDAC risk and mortality	[145,195,196]
	• Oral administration of *P. gingivalis* accelerated progression of PDAC in mice	[197]
	*Prevalence of oral microbiota*	
	• *Fusobacterium* in the gut, pancreatic, and tumor microbiomes of PDAC patients	[85,196,200]

## Data Availability

Not applicable.
